# DNA Associated with Circulating Exosomes as a Biomarker for Glioma

**DOI:** 10.3390/genes11111276

**Published:** 2020-10-29

**Authors:** Manjusha Vaidya, Kiminobu Sugaya

**Affiliations:** Burnett School of Biomedical Sciences, College of Medicine, University of Central Florida, Orlando, FL 32816, USA; Manjusha.vaidya@ucf.edu

**Keywords:** biomarkers, exosomes, cell-free DNA, oncogenes, cancer stem cells, stemness genes

## Abstract

Cancerous and non-cancerous cells secrete exosomes, a type of nanovesicle known to carry the molecular signature of the parent for intercellular communications. Exosomes secreted by tumor cells carry abnormal DNA, RNA, and protein molecules that reflect the cancerous status. DNA is the master molecule that ultimately affects the function of RNA and proteins. Aberrations in DNA can potentially lead a cell to malignancy. Deviant quantities and the differential sequences of exosomal DNA are useful characteristics as cancer biomarkers. Since these alterations are either associated with specific stages of cancer or caused due to a clinical treatment, exosomal DNA is valuable as a diagnostic, prognostic, predictive, and therapeutic-intervention response biomarker. Notably, the exosomes can cross an intact blood–brain barrier and anatomical compartments by transcytosis. As such, the cancer-specific trademark molecules can be detected in systemic blood circulation and other body fluids, including cerebrospinal fluid, with non-invasive or minimally invasive procedures. This comprehensive review highlights the cancer-specific modulations of DNA associated with circulating exosomes that are beneficial as glioma biomarkers.

## 1. Introduction

Cancer is a genetic disease; one of its key attributes is genomic instability. Changes in stemness genes, proto-oncogenes, DNA repair genes, and tumor suppressor genes are the primary drivers of carcinogenesis [1]. In germline and somatic cells, DNA aberrations that are vital characteristics of a cancer genome are marked by mutations, single nucleotide polymorphism (SNP), gene gain, and chromosome gain [2]. The DNA molecules with stage-specific alterations or therapy response modulations are hauled away from the cancer cells in a vesicular transport mediated by exosomes that circulate throughout the body [3]. Exosomes are extracellular nanovesicles (30–100 nm) of late endosomal origin, and they possess a phospholipid bilayer membrane. They are the means of intercellular communication among all types of cells. Exosomes secreted by cancer cells (cancer exosomes) carry pathophysiological properties and act as the mediators of tumor formation, progression, and metastasis [4,5,6]. Mediating horizontal transfer of DNA containing tumor-specific alterations is one of the hallmark roles of exosomes in spreading cancer. These alterations are made by transporting DNA from cancer cells to healthy cells, thereupon transforming the recipients to a precancerous or a cancerous state. However, the exact role of DNA received from the internalized exosome is unclear [7]. In healthy cells, exosomes remove excess cytoplasmic DNA and gene fragments in order to maintain cellular homeostasis. Resulting developments such as increased exosome secretion by the cancer cells and higher amounts of DNA loaded to cancer exosomes compared to their normal counterparts, further elevate the significance of exosomal DNA as a cancer biomarker [8,9,10,11]. Though exosomal RNA and proteins are well-established glioma markers, rigorous studies focused on exosomal DNA began barely six years ago. The research establishing their significance as brain cancer biomarkers is still in early stages. At this time, non-invasive biomarkers for the detection of malignant gliomas, the primary tumors of central nervous system, are still in development. For this reason, we focus our review on the exosome-associated DNA as glioma biomarkers by presenting the highlights of studies conducted by various researchers in the field.

Cancer exosomes transport genomic, cytoplasmic, and mitochondrial DNA as cancer-specific cargo, along with other cellular-blueprint molecules like RNA, proteins, and lipid rafts. All of these biomolecules are reported to be functionally active and pleiotropic in the recipient cell. They participate in modification of the tumor microenvironment, angiogenesis, metastasis, and immunity evasion [12]. It has not yet been clearly established whether the type of cell from which an exosome originates affects the type of cell that can internalize/uptake the exosome. Our knowledge about the mechanisms involved is also limited. Whereas some studies claim to observe no exclusivity between secretion-based exosome type and the target cells, others report that the exosomal membrane plays a role in maintaining the identity of origin and transference compatibility of an exosome. The result of the latter type of study is a preferential transfer and uptake of exosomes. This type of selectivity is helpful in designing special therapies for diseases [13]. Some in-vitro experiments suggest that in a tumor microenvironment, the cancer exosomes likely target other cancer cells over normal cells, thereby serving as selective delivery vehicles [14].

DNA, RNA, and proteins associated with cancer have an ability to predict and identify multiple facets of brain cancer due to their disease-specific modulations. Well-researched, established biomarkers that are found in abundance in exosomes include mRNA, microRNA, long noncoding RNA, and a wide variety of native and cell-derived proteins. Certain characteristics of brain tumors, such as glioblastoma tumor cells’ inability to exit the central nervous system (CNS), together with the exosomes’ ability to cross the BBB to be detected in body fluids, further elevate their significance in brain cancer management [15]. In recent years, rigorous studies regarding exosome-associated DNA have illuminated its cancer-specific properties, leading researchers to add exosomal DNA and its modulations to the list of brain cancer biomarkers. Additionally, a substantial portion of DNA lacking blood plasma cells localizes to exosomes. Moreover, the cancer exosomes possess many times more DNA than their normal counterparts as stated earlier. Therefore, the quantitative analysis of exosome-associated DNA has a significant potential to be used as a cancer biomarker in addition to its modulated nucleotide sequences [11,15].

**Figure 1:**
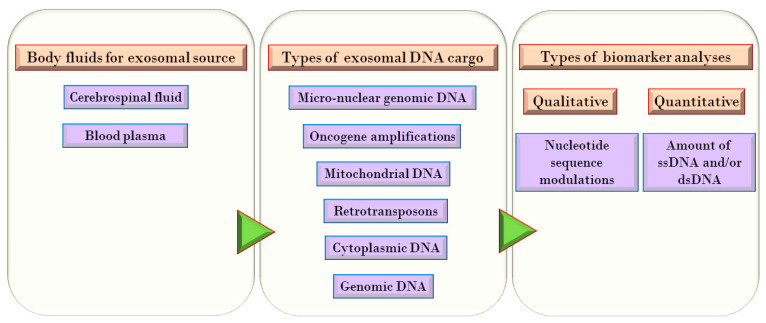
Exosomal DNA as brain cancer biomarkers. The selection of body fluids, the types of DNA and the analyses performed with the exosomal DNA to establish them as prognostic, diagnostic or therapy response biomarkers for the brain cancers.

In glioma, as in any other type of cancer, the accumulation of tumor burden is a prerequisite to detect a tumor, monitor progression and supervise therapeutic response. At some point in time during the accretion process, the tumor cells potentially acquire therapy resistance. This is detrimental to patient survival. Invasive and serial tumor biopsies for histological analyses are used to monitor the progression and effect of treatment. However, they are not only impractical and risky, but ineffective in conveying sufficient information about the tumor due to its heterogeneous characteristics. This creates a need for non-invasive procedures that can detect distinguishing features which accurately reflect tumor status [16]. Therefore, an availability of exosome-associated biomarkers is essential for detecting brain cancers. Identification and analysis of the tumor-specific DNA molecules in body fluid-derived exosomes is an essential clinical tool for diagnosis, prognosis, and prediction of the patient’s response to selected personalized therapies for brain cancers. This is accomplished with a technique known as “liquid biopsy.” This review compiles the details of exosome-associated DNA, genes, and their modulations that have a potential use as biomarkers in brain cancers such as glioma, including its aggressive form Glioblastoma multiforme (GBM). Figure 1 depicts the types, the sources, and the analyses of exosomal DNA used in the research of brain cancer biomarkers.

Primary malignant brain tumors originating within the brain tissue, namely glia and neurons, occur at any stage of life. According to the Central Brain Tumor Registry of the United States, the mortality rate for malignant brain tumor patients has increased to 7.12 per 100,000 in the year 2018 from 4.42 between the years 2012 and 2016 [17]. This dismal situation warrants new and improved techniques for early detection, diagnosis, treatment, and prognosis of brain cancers. Extensive research toward exosome-associated DNA biomarkers is a vital step in the right direction.

### Localization, Types, and Sources of DNA in Exosomes

While current research firmly establishes the modulated exosomal DNA as a cancer biomarker, the mechanism through which the cancer-specific nucleic acid cargo is loaded onto the exosomes is poorly understood. Research findings have partially attributed the presence of severely mutated and aberrated cancer DNA in extracellular vesicles (EV), including exosomes, to the phenomenon of chromoanagenesis such as chromothripsis and kataegis. These genetic events are an important addition to the previous notion that the tumor development occurs with progressive genomic rearrangement. Chromothripsis, a form of genome instability in cancers, is characterized by chromosomal reconstitution and missegregation in specific genomic regions. It is caused by a single catastrophic event due to DNA-damaging agents that trigger a double-stranded DNA break [18,19,20,21]. Kataegis is marked by clusters of base substitutions representing point mutations in specific regions of chromosomes in a cancer cell. Both chromothripsis and kataegis are cancer-specific genome alterations, possibly arising after the collapse of micronuclei in one cell division [22,23]. Upon disintegration of the nuclear membrane, the aberrated micronuclear DNA cargo in the form of SNP, gene gain and chromosome gain, releases into the cytoplasm, ultimately uploading to exosomes. CD63, the exosomal marker and member of the tetraspanin transmembrane 4 superfamily, plays a crucial role in the loading of the DNA to cancer exosomes [24]. The possibility of exosomal localization signals for DNA molecules cannot be ruled out. In GBM, the mRNAs enriched in microvesicles have variants of a “zip-code-like” 25-nucleotide sequence present in 3′UTR that act as a signal for exosome localization. Mutation of the core sequence “CTGCC” of the zip code inhibits the exosomal mRNA gain [25]. A similar sequence may exist for certain DNA molecules that localize to exosomes.

The exosome-associated DNA localizes not only to the interior of the exosome, but also to the exosomal surface. DNase treatment of the intact exosomes reduces the total DNA yield significantly, proving the presence of the surface-associated DNA population [16]. Irrespective of their location, cancer exosomes carry single and double-stranded DNA molecules of various types that include tumor-specific oncogene amplification, aberrated chromosomal DNA, nuclear DNA, micro-nuclear genomic DNA, ribosomal DNA, cell-free DNA (cfDNA), cDNA, mtDNA as well as elevated levels of retrotransposon elements [8,15,26,27]. The type of DNA to be loaded to an exosome is affected by several factors, including the cell treatment. For example, a cell exposed to antibiotics goes into a genotoxic shock, secreting exosomes with predominantly mtDNA on the surface compared to nuclear DNA [28].

The high occurrence of DNA in cancer exosomes is consistent with the higher concentrations of cytosolic DNA found in cancer cells. cGAS (cyclic guanosine monophosphate or cGMP and adenosine monophosphate or cAMP synthase) is a sensor of cytosolic viral DNA that produces cyclic GMP-AMP (cGAMP) signaling molecules to bind to STING (stimulator of interferon response cGAMP interactor 1) proteins and invoke an immune response. The cGAS–cGAMP–STING mediated cytosolic DNA sensing mechanism, with its onco-suppressive effect, is fundamental in controlling malignant transformations and tumor suppression. In cancer, through genetic or epigenetic alterations, cytosolic DNA evades cGAS surveillance, ultimately providing the cell with a proliferative advantage. This possibly leaves higher concentrations of DNA molecules in the cytoplasm to be eventually transported away from the cell via exosomes to maintain cellular homeostasis, thereby evading apoptosis [9,10,29,30].

## 2. Exosomal DNA Modulations as Cancer Biomarkers

Exosomal DNA comprises modulated exome along with introns, non-coding genomic DNA (gDNA) and chromosomal sequences, including transposable elements. Table 1 is a snapshot of the modulated genes and other sequences found in exosomes secreted by various types of primary brain cancers discussed in the review.

### 2.1. NANOG/NANOGP8

Homeobox transcription factor and embryonic stemness (ES) gene NANOG, along with its pseudogene NANOGP8, have critical roles in cancer stem cell (CSC) maintenance, tumor progression, cancer recurrence, metastasis, and therapy resistance [31,32]. Our research revealed differential sequences of these two genes in various types of cancer exosomes, including the ones secreted by GBM. The modulations were found in the form of SNPs, complete absence of certain exon regions, and a 22 base pair insertion in the 3′ UTR of NANOGP8 DNA. The percentage of clones with 22 bp insert was significantly higher in cancer-derived exosomes compared to their normal counterparts. Occurrence of the insert in 3′ UTR region of NANOGP8 is compelling and implies the possibility of its role in translational modification of cancer. The size of this insert is also significant, indicating its prospective role as a microRNA-annealing site. The microRNAs are typically 19–25 nucleotides long and their annealing to 3′UTR region of an mRNA has an expression regulatory role in cancers [27,33]. This study needs further investigation—using cancer exosomes derived from body fluids of enough number of patients—to gain statistical power. Because CSCs are treatment-resistant cells responsible for the cancer recurrence and metastasis, modulated exosomal DNA of NANOG and NANOGP8 has a potential in assessing the efficacy of clinical therapy [34].

### 2.2. SOX2

Another ES gene, SRY-box transcription factor 2 (SOX2), is associated with brain cancers and maintenance of CSC, specifically, in GBM and pediatric brain tumors, including glioma. Elevated SOX2 expression levels positively correlate to malignancy grades, metastasis, drug resistance and poor survival. These attributes make SOX2 an ideal prognostic biomarker [35,36,37,38]. At a phenotypic and molecular level, the pediatric brain tumors differ from adult brain tumors such as GBM [38]. However, it is noteworthy that the CSC in both, adult, and the pediatric brain tumors, retain the functionality of pluripotency-biomarkers such as NANOG, SOX2, and OCT4 [39,40]. In our research to analyze the differential sequences of ES genes associated with cancer exosomes, we found many-fold more SNPs and also a cancer-specific SNP associated with GBM-derived exosomes. A SOX2 SNP, rs11915160, at chr3:181713783 (A > C) evaluated for susceptibility to breast cancer was detected in exosomal SOX2 DNA of GBM, and GBM stem cells. Body fluid-derived exosomal analysis of this particular SNP will provide more robustness as a brain cancer biomarker. Additionally, the neural stem cell, CSC and the cancer exosomes also carried the SOX2 3′UTR region that contains the binding sites for microRNAs miR-126 and miR-522 [41]. The presence of miRNA annealing sites of the SOX2 gene in exosomal DNA is significant because miR-126-3p is known to sensitize GBM to TMZ by targeting SOX2. and miR-522 has been shown to play a role in many human cancers [42,43].

### 2.3. EGFR

Amplification of epidermal growth factor receptor EGFR and its mutant EGFRvIII (deletion: amino acid 6-273) is found in more than 50% GBM patients, indicating a worse prognosis as compared to the patients without the mutation. EGFRvIII confers the tumor with better viability compounded by invasiveness, stemness and angiogenesis, making the gene and its transcription product a well-suited candidate for targeted therapy [44]. Circulating exosomes from GBM patient’s plasma have detectable EGFRvIII DNA deletions that are reflective of the mutation in gDNA. The finding makes the exosomal EGFRvIII a diagnostic, prognostic, and therapy marker for anti-EGFRvIII vaccine and other EGFRvIII targeted therapies using tyrosine kinase inhibitors (TKIs) or antibodies. The complete absence of circulating EGFRvIII DNA in post-surgical patients is an indicator of absolute tumor resection and an effective biomarker for tumor status monitoring [45].

### 2.4. MGMT

O-6-methylguanine-DNA methyltransferase (MGMT) gene encodes a DNA repair protein that has a role in cellular defense against mutagenesis and alkylating agent-induced toxicity. MGMT promoter hypermethylation, an epigenetic event and a mark of GBM present in approximately 40% of the patients, results in the loss of gene function leading to an inefficient DNA repair. Exosome-associated gDNA in GBM patients reveals MGMT gene sequences [3]. Elevated levels of exosome-associated MGMT DNA with a methylated promotor detected in GBM patient’s serum correspond to an improved response to alkylating agents such as Temozolomide (TMZ). It also indicates a slower progression of the tumor, thereby becoming an only known prognostic and a therapy response biomarker [46]. Independent of the promotor methylation status, some gliomas have MGMT gene alterations and genomic rearrangement leading to MGMT overexpression and TMZ resistance. Tumor-derived exosomes carry these DNA fragments which act as the biomarker of tumor recurrence in TMZ-treated patients [47].

### 2.5. IDH1/IDH2

Mutations in key metabolic enzymes Isocitrate dehydrogenase 1 and 2 (IDH1 and IDH2) are defining features of primary brain cancers that include low-grade gliomas and secondary GBM. IDH1 mutation at a single amino acid residue arginine to histidine (R132H: NM_001282386.1 (IDH1):c.395 G > A) is present in approximately 80% of these types of brain cancers. IDH2 mutations R140Q (NM_001289910.1 (IDH2):c.263 G > A) and R172K (NM_001289910.1 (IDH2):c.359 G > A) have prognostic impact. Where R140Q may bestow a relatively favorable outcome, R172K confers a grim prognosis with higher recurrence and low survival rates [48,49]. In *IDH1/2* mutation-containing gliomas, the genetic modifications are reported to forerun other genetic alterations. This implies the role of *IDH1/2* mutation as the effector of the cancer initiation. In the light of these findings, a GBM-specific *IDH1*^G395A^ mutation detected in exosomal gDNA of patients’ peripheral blood is of utmost value as a molecular marker to evaluate diagnosis and prognosis of low and high-grade gliomas [3].

### 2.6. mtDNA

Mitochondria are known to migrate to neighboring cells via tunneling nanotubes, moving mtDNA along with them. In a paracrine fashion, mtDNA is found to move from one cell to another as an exosomal cargo. Cancer cells are known to contain limited number of mitochondria due to the Warburg effect involving tumor cells’ shift from oxidative metabolism to a glycolytic, non-aerobic energy metabolism. However, the cancer cell mitochondria possess more DNA coding for bioenergetics genes [50]. Though the mitochondrial heteroplasmy reflecting mtDNA aberrations is an accumulation of the errors happened during rapid proliferation of tumor cells, the exosomes harboring full mitochondrial genome with these modulations might represent a mode of transportation to restore metabolism in the recipient cells. Heteroplasmic mutations gathered in mtDNA over time that deteriorate the energy production capacity of a cell are important in cancer. Cellular energetics is a limiting factor in cancer cell survival and proliferation. Modulated mtDNA is a means of energy optimization to suit this changing microenvironment [51,52,53]. Additionally, during clonal tumor growth, differential mtDNA sequences representing heteroplasmy may accumulate into a homoplasmic state. In such instances, the tumor-derived mtDNA can potentially act as a stable tumor marker. In grade III astrocytomas and GBM, mitochondrial displacement loop (D-loop) is affected showing a sequence variability [54]. In GBM, during differentiation, mtDNA copy-number gain is associated with initiation and maintenance of tumorigenesis [55]. Among different types of deletions in mtDNA, a large deletion of 4977 base pairs (nucleotide 8.470 and 13.447), known as common deletion, resulting in partial or complete removal of five tRNA genes, as well as Fo-F1 ATPase, C1, and complex IV subunits, is found in several types of cancers. mtDNA containing common deletion can potentially be found in enriched levels in the exosomes of cancer patients’ circulating blood, thereby becoming a cancer marker [56]. Higher quantities of altered mtDNA are detected in GBM exosomes over normal astrocyte-derived exosomes. DNA for human mitochondrial encoded NADH dehydrogenase subunit 1 is found in the exosomes of astrocyte and GBM origin. Therefore, migration of mtDNA via cancer exosomes can be a powerful diagnostic biomarker [57]. Genotoxic shock induced by cancer treatment may have the potential to send mtDNA on the surface of a cancer exosomes just as the antibiotic-induced effect does. If investigated for brain cancer exosomes, this type of mtDNA on exosomal surface can become a molecular marker attesting to the effects of chemotherapy drugs [58]. Radiation exposure is found to elevate the mtDNA levels in exosomes secreted by human fibroblast cells [59]. Based on these findings, a quantitative analysis of mtDNA, secreted by tumor exosomes after radiotherapy, may shed light on the effect of this treatment. Not just quantitative increase, but also the nucleotide sequence modulations of mtDNA can be used as a brain cancer biomarker. In our unpublished data, GBM-derived exosomes had many-fold more SNP in the D-loop region (nucleotide position 321–496) as compared to their normal counterpart. These results warrant further research to establish the SNPs as biomarkers.

### 2.7. CDKN2a, PTEN and TP53

Tumor protein p53 (TP53) is a tumor suppressor protein that regulates expression of target genes involved in vital cancer-related cellular processes such as DNA repair, cell cycle arrest and apoptosis. Mutations in TP53 are associated with a variety of cancers, including brain cancers. Sonic-Hedgehog medulloblastoma brain tumor is reported to have a T53 mutation and 24 tumor-specific single nucleotide variations [21,60]. More than 60% of grade II astrocytomas and GBM have a loss of TP53 locus and the retained allele is mutated. The majority of GBM have no wild type TP53 [61]. TP53 protein expression is downregulated in GBM exosomes [62]. Though not qualified to be a typical exosomal biomarker, a remarkable absence of TP53 gDNA in cancer exosomes–derived from GBM–is attributed to the loss of heterozygosity of chromosome 17 at position p13.1. Similarly, loss of heterozygosity of chromosome 9 at p21.3 and chromosome 10 at q23.1 is reflective of the absence of the gene fragments of CDKN2a and PTEN respectively, from cancer exosomes. The absence of CDKN2a, PTEN and TP53 in exosomal gDNA is prominent and interesting because of the presence of other GBM-relevant gDNA sequences such as EGFR, CDK4, AKT3, ERBB2m and MDM2 in GBM exosomes [3].

### 2.8. ERBB2, CDK4, AKT3, MDM2, and RB1

Exosomes derived from the peripheral blood samples of human glioma patients are reported to carry GBM-specific gDNA of ERBB2, CDK4, AKT3, MDM2, and RB1 genes [3]. erb-b2 receptor tyrosine kinase 2 (ERBB2), a member of the epidermal growth factor receptor family, along with genes such as cyclin dependent kinase 4 (CDK4), AKT serine/threonine kinase 3 (AKT3) and TP53 function inhibitor- murine double minute 2 (MDM2), are amplified and overexpressed in gliomas, glioblastoma and meningioma [63,64,65,66]. Retinoblastoma Tumor Suppressor Protein (RB1) is a marker for GBM therapeutic efficacy [68]. It is noteworthy that in breast cancer, disruption of RB pathway is associated with an increase in the invasiveness of ERBB2 overexpressing cells [67]. This type of cancer exosome DNA cargo, along with selection of an appropriate profiling technology such as multiplex PCR, can potentially serve as a diagnostic biomarker “panel” for a wide range of brain tumors, including gliomas.

### 2.9. c-Myc and POU5F1B

Medulloblastoma and GBM serum-derived exosomes contain amplified sequences of the proto-oncogene c-Myc reflecting the copy number gain at the genomic level. 10–25-fold more c-Myc specific sequences, including full introns, are detected in the medulloblastoma-derived exosomes compared to the exosomes secreted by fibroblasts. The medulloblastoma exosomal gDNA, along with amplified c-Myc, also contains elevated levels of POU5F1B that sits adjacent to c-Myc on 8q24. POU5F1B, a pseudogene of POU class 5 homeobox 1 transcription factor (OCT4), plays a significant role in the aggressiveness of carcinogenesis, including gliomas. The amplified POU5F1B gene is an indicator of poor prognosis and a useful biomarker in brain cancers [8,69,70].

### 2.10. PD-L1

Cancer progression is possible due to the suppression and evasion of immune response by tumor cells. Exosomal DNA, through regulation of cytoplasmic DNA sensing STING pathway, acts as a regulator of tumor immunity. Exosomal DNA exchange between tumor cells and dendritic cells regulate anti-tumor immunity [71]. Characteristics of glioma, especially GBM’s aggressive invasion, metastasis, and recurrence, are attributed to its powerful immunosuppression. Programmed death ligand-1 (PD-L1) binds to programmed cell death protein-1 (PD-1) to inhibit the T-cell function leading to immune evasion by GBM. Enriched levels of PD-L1 DNA found in serum and plasma exosomes of GBM patients reflect the high expression of PD-L1 in tumor cells. Additionally, the elevated levels of PD-L1 DNA in circulating exosomes correlate with tumor volume, becoming a potential marker for monitoring tumor size and progression [72].

### 2.11. L1 and HERV

Transposable elements (TE), through insertion in the genome, cause genomic and genetic variation. Through their deletion, insertion and recombination events, the TE can provide a cis-regulatory element. These modulations provide promoter, enhancer and transcription factor binding sites thereby manipulating host genome and gene expression. Their demethylation and reactivation is associated with cancers involving activation of an innate antiviral response. Exosome-associated tumor-specific TE can be potentially important biomarkers for immunotherapy [76,77]. Endogenous retrotransposable element long interspersed element-1 (LINE-1 or L1) transposition is not known to occur commonly in primary GBM, glioma or other brain cancers. L1 may act as a very rare insertional mutagen in secondary GBM [73,74]. However, medulloblastoma exosomes are found with enriched levels of L1 DNA. Similarly, transcriptionally active retrotransposon human endogenous retrovirus K (HERV-K) is associated with many cancers, but do not seem to have a role in brain cancers [75]. Yet, HERV-K DNA, like L1, is abundant in cancer exosomes of medulloblastoma. Trace amounts of both retrotransposons are found in GBM exosomes as well [8]. This suggests that the expressions of these two elements may not have a role in brain cancers. Though unsuitable targets for brain cancer therapy, their exosome-associated DNA proves to be potentially significant as a diagnostic and a tumor progression biomarker.

## 3. Conclusions

The research focused on finding suitable exosomal DNA biomarkers for glioma management is an ongoing endeavor with many challenges. After crossing the discovery and validation stages, the exosomal DNA modulations discussed in this review have tremendous potential to be adopted and applied as clinical biomarkers in the near future. Many more cancer exosome-associated DNA modulations need to be researched and affirmed for biomarker use. Some of the important DNA aberrations such as 1p/19q co-deletion and TERT promoter mutation useful as diagnostic, prognostic and predictive brain cancer biomarkers have not been studied in exosomes. Similarly, glioma-associated SNPs and modulations in 8q24.21 need analysis in glioma-secreted exosomes [78]. Even though the EV cargo, including the DNA associated with cancer exosomes, works toward alleviating the need for brain cancer biomarker-acquisition, its establishment as a clinical biomarker needs empirical evidence as well as bench work and theoretical analyses. To qualify as a brain cancer biomarker, the exosomal DNA modulations must be able to provide specific clinical information pertaining to the disease and the treatment. Its analytical rationality needs to be assessed in order to meet the stringent criteria required for translation into clinical validity. To conclude, following the guidelines set by the International Society for Extracellular Vesicles (ISEV), making the exosomal DNA a meaningful biomarker will need discovery experiments in the research laboratories as well as pharmacogenomic testing to invoke confidence in their ultimate usage as a clinical tool benefitting brain cancer patients [79,80].

## Figures and Tables

**Table 1:** Modulated genes and transposable element DNA sequences associated with brain tumor-secreted exosomes. The details of each type of DNA and their use as brain cancer biomarkers are compiled in the following section.

Gene/DNA	Type of Modulation in Exosomal DNA	Reference
*NANOG/NANOGP8*	SNP, insertions	[27,31,32,33,34]
*SOX2*	SNP, breast cancer specific SNP	[35,36,37,38,39,40,41,42,43]
*EGFR*	amplification, mutation	[44,45]
*MGMT*	methylated promoter, gene alterations, genomic rearrangements	[3,46,47]
*IDH1/IDH2*	mutations	[3,48,49]
mtDNA	SNP, elevated quantities	[50,51,52,53,54,55,56,57,58,59]
*TP53*	gDNA absent (loss of heterozygosity of chromosome 17 at position p13.1)	[3,21,60,61,62]
*CDKN2a*	gDNA absent (loss of heterozygosity of chromosome 9 at p21.3)	[3]
*PTEN*	gDNA absent (loss of heterozygosity of chromosome 10 at q23.1)	[3]
*ERBB2*	GBM-specific gDNA	[3,63,64,65,66,67]
*CDK4*	GBM-specific gDNA	[3,63,64,65,66]
*AKT3*	GBM-specific gDNA	[3,63,64,65,66]
*MDM2*	GBM-specific gDNA	[3,63,64,65,66]
*RB1*	GBM-specific gDNA	[3,67,68]
*c-Myc*	amplified sequences of gDNA (including full introns)	[8,69,70]
*POU5F1B*	elevated levels of gDNA, sits side by side to c-Myc on chromosome 8q24.	[8,69,70]
*PD-L1*	elevated levels of DNA	[71,72]
L1	enriched levels of transposable element DNA	[8,73,74]
HERV	enriched levels of transposable element DNA	[8,75]
